# Taste substance binding elicits conformational change of taste receptor T1r heterodimer extracellular domains

**DOI:** 10.1038/srep25745

**Published:** 2016-05-10

**Authors:** Eriko Nango, Shuji Akiyama, Saori Maki-Yonekura, Yuji Ashikawa, Yuko Kusakabe, Elena Krayukhina, Takahiro Maruno, Susumu Uchiyama, Nipawan Nuemket, Koji Yonekura, Madoka Shimizu, Nanako Atsumi, Norihisa Yasui, Takaaki Hikima, Masaki Yamamoto, Yuji Kobayashi, Atsuko Yamashita

**Affiliations:** 1 RIKEN SPring-8 Center, 1-1-1, Kouto, Sayo, Hyogo, 679-5148, Japan; 2 Research Center of Integrative Molecular System (CIMoS), Institute for Molecular Science, National Institute of Natural Sciences, 38 Nishigo-Naka, Myodaiji, Okazaki, Aichi, 444-8585, Japan; 3 Department of Functional Molecular Science, The Graduate University for Advanced Studies (SOKENDAI), 38 Nishigo-Naka, Myodaiji, Okazaki 444-8585, Japan; 4 Food Research Institute, NARO, 2-1-12, Kannondai, Tsukuba, Ibaraki, 305-8642, Japan; 5 Graduate School of Engineering, Osaka University, Suita, Osaka, 565-0871, Japan; 6 Okazaki Institute for Integrative Biosciences, Okazaki, Aichi 444-8787, Japan; 7 Graduate School of Medicine, Dentistry and Pharmaceutical Sciences, Okayama University, 1-1-1, Tsushima-naka, Kita-ku, Okayama, 700-8530, Japan

## Abstract

Sweet and umami tastes are perceived by T1r taste receptors in oral cavity. T1rs are class C G-protein coupled receptors (GPCRs), and the extracellular ligand binding domains (LBDs) of T1r1/T1r3 and T1r2/T1r3 heterodimers are responsible for binding of chemical substances eliciting umami or sweet taste. However, molecular analyses of T1r have been hampered due to the difficulties in recombinant expression and protein purification, and thus little is known about mechanisms for taste perception. Here we show the first molecular view of reception of a taste substance by a taste receptor, where the binding of the taste substance elicits a different conformational state of T1r2/T1r3 LBD heterodimer. Electron microscopy has showed a characteristic dimeric structure. Förster resonance energy transfer and X-ray solution scattering have revealed the transition of the dimerization manner of the ligand binding domains, from a widely spread to compactly organized state upon taste substance binding, which may correspond to distinct receptor functional states.

Taste sensation is evoked by specific interactions between taste substances and taste receptors residing in the plasma membranes of the taste cells in taste buds in the oral cavity^1^,^2^. One of these receptors is the taste receptor type 1, the T1r family, which is evolutionarily conserved in vertebrates, including fishes, birds, and mammals^3^. The heterodimer of T1r2 and T1r3 recognizes sweet taste substances such as sugars and artificial sweeteners, while the heterodimer of T1r1 and T1r3 recognizes umami taste substances such as l-glutamate^4^,^5^,^6^.

The T1r family proteins belong to the class C G-protein coupled receptor (GPCR) family^7^,^8^. The class C GPCR members function as constitutive homo- or heterodimers in the physiological state. The class C GPCR structure is characterized by the presence of a large extracellular domain upstream of the hepta-helical transmembrane region, which is commonly found among GPCRs. The extracellular domain consists of the ligand binding domain (LBD), responsible for primary agonist binding, followed by the cysteine rich domain (CRD), which mainly serves as a linker between the LBD and the transmembrane region (Fig. 1a). Ligand binding at the extracellular domain results in receptor activation and signal transmission to the heterotrimeric G-protein in the cytosol^7^,^8^. The receptor activation mechanism of the class A GPCR members, consisting solely of the transmembrane region, has been considered to occur via agonist binding, which changes the conformational dynamics of the protein by lowering the transition energy between the different states, and results in the transition towards the active-state conformation^9^. In contrast, the conformation of the transmembrane region of the class C GPCRs is considered to be allosterically regulated by agonist binding to the extracellular LBDs, probably through their conformational changes^10^,^11^,^12^,^13^. Accordingly, in the case of T1r, the major taste substances, including sugars and l-glutamate, are considered to target the LBD of T1r heterodimer^14^, and thus consequently induce the conformational change of the LBD.

Due to the lack of structural information of T1r receptors, their functional mechanisms have so far been conjectured from the crystallographic observation on the other class C GPCR members. Crystal structures of the LBDs of metabotropic glutamate receptors (mGluRs) and GABA_B_ receptor (GABA_B_R) revealed the bilobal architecture; namely, the Venus-flytrap domain (VFTD), in which an agonist binds to the cleft between the two lobes, LB1 and LB2^15^,^16^. Upon agonist binding, two types of conformational change were observed on the LBDs (Supplementary Table S1). One is the domain closure within the protomer, at the cleft between LB1 and LB2, which is referred to as the open- and closed-conformation. The other is the change between the two different forms of the dimer arrangement: the compact state with a smaller torsion angle (< ~ −30°) rotated around the axis between the LB1s of the two protomers (Fig. 1a), interpreted as the active (A)-state, and the widely spread states with a larger torsion angle (> ~ −50°), interpreted as the resting (R)-state (Supplementary Fig. S1)^15^. This view is basically compatible with the observations by Förster resonance energy transfer (FRET) of labeled mGluRs^12^,^13^. However, the actual conformations of the LBDs in the physiological state are still unknown, because available structural information is limited to those obtained in crystalline spaces. In fact, the dimer arrangements of homodimeric mGluR LBDs so far observed there were various regardless of the types of bound ligands, such as agonists, antagonists, or ligand-free^15^,^17^,^18^ (Supplementary Table S1), and a previous study also pointed out the possibility of biased trapping of certain conformations among dynamic conformational equilibrium by the crystal packing^18^. Moreover, a crystallographic analysis of the LBD of GABA_B_R GBR1/GBR2 heterodimer revealed another mode of conformational change, where little significant dimer rearrangement is observed among apo-, antagonist bound-, and agonist bound-states^16^ (Supplementary Fig. S1 and Table S1). The current situation makes it obscure what kind of conformational change at the T1r LBD heterodimer is elicited by taste-substance binding.

So far, not only the structural analyses but also the molecular functional analyses using the purified protein of T1rs have been completely hampered, due to the difficulties in the heterologous expression and purification even for the partial regions such as the LBD^19^. In this study, we found that the LBDs of T1r2 and T1r3 of medaka fish (*Oryzias latipes*, also known as Japanese rice fish), a representative model organism in vertebrates, can be expressed heterologously as a properly folded and functional heterodimeric protein, thus enabling various biophysical analyses. The solution-state structure analyses revealed the conformational change upon the taste substance binding to the T1r, under the condition devoid of any constraint derived from crystal packing.

## Results

### The ligand-binding domains of the T1r heterodimer from medaka fish exhibit proper recombinant expression

**Figure 1:**
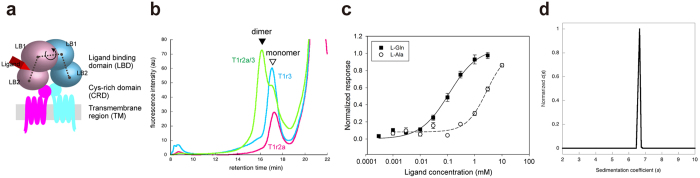
Taste Receptor T1r Proteins from Medaka Fish (mf). (**a**) Schematic drawing of the overall architecture of class C GPCR, where the codebook vector of each domain in LBD (gray dot) and the protomer torsion angle (the arrow) were depicted. (**b**) FSEC analysis of mf T1R2aLBD, mf T1R3LBD, and co-expression of the T1R2a and T1r3 proteins. (**c**) Dose-response curves for l-alanine and l-glutamine by the full-length mf T1r2a/T1r3 receptor in HEK293 cells. The error bars are ± SEM of 4–34 independent determinations. (**d**) The *c*(*s*) distribution of the purified mf T1r2a/3LBD, obtained from the data analysis of SV-AUC experiments.

Chemosensory receptors, such as olfactory receptors and pheromone receptors, are known to have the specific chaperone systems in their native chemosensory cells, and the proper surface expression of the receptors in heterologous cells are only achieved in the presence of the systems^20^,^21^,^22^,^23^. However, in the case for taste receptor T1rs, such specific chaperone systems are unknown^20^, and indeed recombinant expression of mouse- or human-T1rLBDs displayed failure of folding and localization^19^. Thus we first performed extensive screening of T1r genes and expression conditions to find those exhibiting proper expression, using fluorescence-detection size-exclusion chromatography (FSEC)^19^,^24^. Most of the genes from different species, as well as the numerous variations of the expression conditions including host cells, resulted in unsuccessful protein production, as reported previously^19^. Nevertheless, the LBDs of T1r2a and T1r3 from medaka fish (mf)^25^ showed good secretion and a sharp FSEC elution peak corresponding to the dimer species, indicating proper folding and oligomerization, only when they were co-expressed in insect cells (Fig. 1b). mf T1r3LBD showed fair conservation with mammalian T1r3LBD (~37% similarity). On the other hand, LBD of mf T1r2a, one of the three T1r2 subtypes in medaka fish, showed moderate conservation with both mammalian T1r1LBD and T1r2LBD (37~39% similarity), reflecting the fact that the mfT1r2a/T1r3 heterodimer responds to a wide array of l-amino acids, but not to sugars or artificial sweeteners such as saccharin^26^.

Dose-response measurement of the full-length mf T1r2a and mf T1r3 heterodimer cloned in this study confirmed the similar EC_50_ value for l-alanine (2.70 ± 1.20 mM) to that reported previously^26^, and revealed an even higher affinity to l-glutamine, with the EC_50_ value of 100 ± 26.0 μM (Fig. 1c and Supplementary Table S2). Because medaka fish reportedly show preferences to the foods containing amino acids^27^, l-glutamine and alanine are considered to serve as taste substances to medaka fish. Therefore, the mf T1r2a/mf T1r3-LBD heterodimer and its amino-acid binding are expected to serve as the first molecular platform to assess the general structural and functional properties T1r LBD heterodimer, including those for taste substance binding.

### T1r2aLBD and T1r3LBD form a stable heterodimer

mf T1r2aLBD and mfT1r3LBD were successfully co-purified after recombinant expression in insect cells (Supplementary Fig. S2). The purified protein exhibited a monodisperse distribution as confirmed by a sedimentation velocity analytical ultracentrifugation (SV-AUC) analysis. In the obtained *c*(*s*) distribution, a single peak with an estimated molecular weight of 108.5 kDa was observed (Fig. 1d). This result clearly indicated that the purified T1r2aLBD and T1r3LBD (53.5 and 55.2 kDa respectively, as estimated from the amino acid sequences) exclusively formed a stable heterodimer.

**Figure 2:**
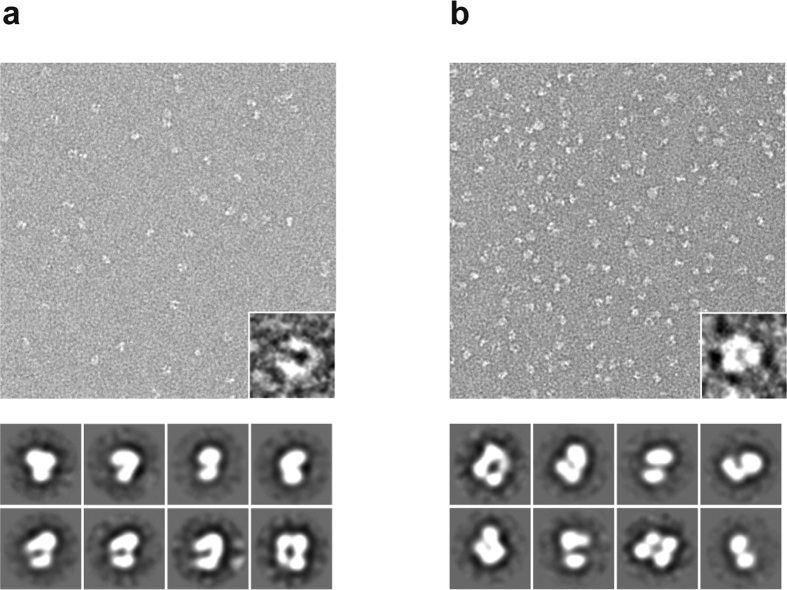
Electron microscopic observation of T1r2a/3LBD. The top panels are negative-staining raw particle images of the purified T1r2a/3LBD, with close-up views of representative particles in the insets. The bottom panels are the representative two-dimensional class averages of particles. (**a**) The l-glutamine-bound state. (**b**) The ligand-free state.

To visualize the structural organization of T1r2a/3LBD, we performed the electron microscopic observation of negatively stained T1r2a/3LBD (Fig. 2). The particles exhibit the presence of two segments, most likely corresponding to each protomer of T1r2a and T1r3 LBD proteins, both in the presence or absence of a taste substance l-glutamine. Two-dimensional averages of T1r2a/3LBD particle exhibited a bilobal feature, characteristic to VFTD. The approximate particle sizes were observed as ~95 × ~75 Å, which agrees with those for other class C GPCR LBD dimer structures. These observations indicated that the manner of dimerization of T1r2a/3LBD is similar to that observed on LBDs of other class C GPCRs, such as mGluRs.

### Conformational change of the ligand binding domains upon taste substance binding

So far, all the structural analyses of other class C GPCR LBDs were performed by crystallography, using deglycosylated samples in many cases^15^,^16^,^17^,^18^. In this study, we performed multiple structural analyses in both the presence and absence of a taste substance, using the glycosylated protein sample (Supplementary Fig. S2) in solution, which is closer to a physiological condition without any constraint derived from crystal packing.

**Figure 3:**
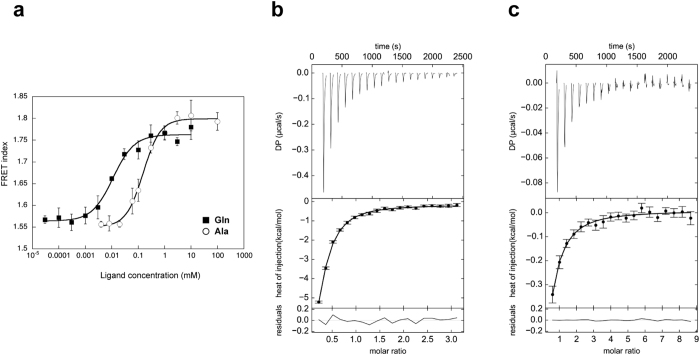
Conformational change of T1r2a/3 LBD upon taste substance binding. (**a**) Dose-dependent FRET signal changes of the T1r2aLBD-Cerulean and T1r3LBD-Venus heterodimer for taste substance binding. The error bars are ± SEM of 3 independent determinations. (**b,c**) l-Glutamine (**b**) and l-alanine (**c**) binding to mfT1r2a/3LBD, measured by isothermal titration calorimetry. The upper and lower panels show the raw data and the integrated heat signals upon ligand injection, respectively, with binding isotherms fitted assuming 1 ligand: 1 heterodimer binding.

We first assessed whether the binding of a taste substance to the T1r LBD induces a conformational change of the protein. To answer this question, the T1r2a/3LBD heterodimer fused with a fluorescence protein, either Cerulean (a CFP variant) or Venus (a YFP variant)^28^, at their C-termini was subjected to Förster resonance energy transfer (FRET) analyses. In the presence of the taste substances l-glutamine and l-alanine, the labeled T1r2a/3LBD exhibited elevated FRET signals (Fig. 3a). The EC_50_ values for the FRET signal changes upon the l-glutamine and l-alanine titration were 12.7 ± 2.7 and 168 ± 19.0 μM, respectively (Supplementary Table S2). We confirmed that the FRET signal rise accompanies the taste substance binding, as the EC_50_ values for the former are close to the *K*_d_ values of l-glutamine and l-alanine to the non-labeled T1r2a/3LBD analyzed by isothermal titration calorimetry (Fig. 3b,c and Supplementary Table S2). These results indicated that taste substance binding to the T1r2a/3LBD induces a conformational change of the protein, in most likely which the C-termini of the LBDs of the two protomers come closer to each other.

The order of the EC_50_ values of two amino acids was coincident with that for the receptor responses as observed above. Although the EC_50_ values for the FRET change by the labeled-LBD and the response by the full-length receptor have 8~16 fold differences, such deviations were often observed on the other receptors when the different assay conditions/methods were used, because the ligand efficacies are affected by the intrinsic receptor characteristics, the downstream signaling pathways, and so on^29^. For example, mGluR1 exhibited the *K*_d_ of 1.3 μM for the l-glutamate binding to the purified LBD measured by fluorescence change^30^, while EC_50_ of 4.6~22 μM for the l-glutamate response measured by Ca^2+^ -activated Cl^−^ current in a *Xenopus* oocyte expression system (3.5~17 fold differences)^31^. Therefore, the results observed in this study suggested that the conformational transition is relevant to the receptor responses.

**Figure 4:**
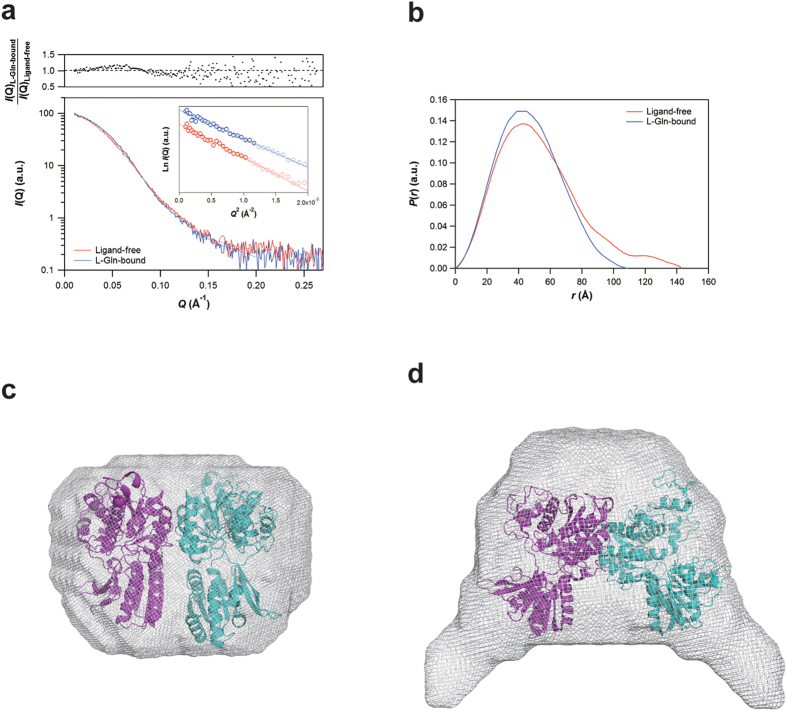
Overall shapes of T1r2a/3 LBD in ligand-free and l-glutamine-bound states revealed by SAXS. (**a**) SAXS curves of the ligand-free (red) and l-glutamine-bound (blue) forms of T1r2a/3 LBD. The inset indicates the Guinier plots of the ligand-free (red) and l-Gln-bound (blue) forms of T1r2a/3 LBD, for which the Guinier analyses were conducted by using the *Q* range (highlighted data points in the inset) from 0.01003 Å^−1^ to *Q*_max_ < 1.3/*R*_g_. (**b**) Pair distribution functions, *P*(*r*), of the ligand-free (red) and l-gln-bound (blue) forms of T1r2a/3 LBD. Low-resolution models of the l-glutamine bound state (**c**) and the ligand-free state (**d**). The representative models were presented as smooth molecular envelopes, onto which the high-resolution models of the glutamate-bound A-state structure of mGluR1LBD (PDB 1EWK) and the ligand-free R-state of mGluR1LBD (PDB 1EWT) were superimposed. The theoretical SAXS curves of the restored models are shown by solid lines in panel (**a**) and are in good agreement with the experimental curves.

To analyze the conformational changes in further detail, the non-labeled T1r2a/3LBD, in the presence or absence of l-glutamine, was subjected to small-angle X-ray scattering (SAXS) analyses (Fig. 4). The molecular mass estimated on the bases of the forward scattering (121~123 kDa) as well as the Porod volume (144~150 kDa) was nearly constant irrespective of the presence of l-glutamine (Supplementary Table S3), exhibiting a fair agreement with the sum of those for T1r2aLBD and T1r3LBD determined by mass spectroscopy and SDS-PAGE (127 kDa; Supplementary Fig. S2).

The radius of gyration (*R*_g_) in the presence of glutamine (37.0 ± 0.5 Å) was smaller than that of the ligand-free protein (39.8 ± 0.6 Å; Supplementary Table S3). This difference in the *R*_g_ values amounted to ~7.6%, a comparable value to those analyzed by the previous SAXS measurements on the nucleotide-mediated conformational change of the myosin head domains (4.5~5.9%)^32^ or the open-close transition of phosphoglycerate kinase during catalysis (~8.0%)^33^. This result clearly indicates that a significant conformational change occurs between the two states.

The radical conformational switching was also supported by the pair distance distribution functions, *P*(*r*), which exhibited a ~30 Å reduction in the maximum dimension of particles (*D*_max_) upon glutamine binding (Fig. 4b and Supplementary Table S3). Judging from the crystal structures of other class C GPCR LBDs, the observed particle size difference seems not be solely attributed to the conformational change within a protomer, but to the rearrangement of the dimerization. For example, the open- (O) to close (C) transitions within the same dimerization state, such between PDB IDs 3KS9 to 1EWK (mGluR1) or 4MQE to 4MS3 (GABA_B_R), results less than 3 Å differences, while the R- to A-state transitions of the dimerization state, such between 1EWT to 1EWK (mGluR1), makes more than 10 Å differences (Supplementary Fig. S3 and Table S1). The radical change in the *D*_max_ of the ligand-free state is a consequence of the decrease and the increase in the probabilities at pair lengths of 30~50 Å and 80~140 Å, respectively. This rearrangement of the pair distribution produced a point of intersection between the ligand-free and the glutamine-bound *P*(*r*)s at a pair length of ~60 Å, which is closer to those between the R- and A-states *P*(*r*)s (~60 Å) than those between O-C transition *P*(*r*)s (~50 Å) (Supplementary Fig. S3). The observation further supported the dimerization rearrangement of T1r2a/3LBD heterodimer upon taste substance binding.

The different overall shapes of the ligand-free and the glutamine-bound T1r2a/3LBD were confirmed by restoring low-resolution models from the SAXS data. The l-glutamine bound T1rLBD took a flat round shape, resembling an A-state structure of class C GPCR LBDs (Fig. 4c). On the other hand, the ligand free T1rLBD displayed a change of the shape into a more extended state, which is reminiscent of that proposed for the A- to R-state transition (Fig. 4d). The results also implied that there at least is a dimer rearrangement between the two states.

All together, the results in this study indicated that the conformational equilibrium of T1r2a/3LBD moves from the widely spread state toward the compact state upon taste substance binding. The directions of conformational changes are generally consistent with those observed on the other class C GPCR LBD structures, and likely more resemble to that observed on mGluR-LBD with dimer rearrangement, rather than that observed on GABA_B_R without significant dimer rearrangement.

## Discussion

This study firstly presented the biophysical properties of the ligand binding domains of a taste receptor T1r heterodimer and their binding to a taste substance. All the analyses in this study were performed in solution using the glycosylated protein sample, which is closer to a physiological condition compared to those with the deglycosylated samples in the crystalline states, therefore the observed properties are expected to well reflect the native characteristics of T1rLBD.

The study revealed two different conformational equilibriums dependent on the existence of a taste substance, the widely spread state and the compact state. In particular, the results clearly indicated the correlation between the taste-substance binding (analyzed by ITC) and the conformational transition (analyzed by FRET and SAXS) of LBD, as both were occurred in the same concentration range of the ligands. Therefore the observations in this study provided solid evidence that the ligand binding induces the conformational change of the LBD, which has been long assumed but not yet actually demonstrated. Each LBD conformation may correspond to a distinct receptor functional state of T1r, and/or the transition between them might induce the transition between distinct functional states. The labeled points for detection of the conformational change in the FRET analysis were the C-termini of the LBDs, which are the direct connection points toward the CRD, followed by the transmembrane region. The crystallographic structure of the transmembrane region of mGluR1 revealed the tight interaction between the linker region connecting the CRD to the transmembrane region, and the extracellular loop 2 in the transmembrane region connecting to the transmembrane helix III, which is reportedly important for receptor activation in class A GPCRs^34^. Based on these observations, the results suggest a possible picture of the taste signal transduction by T1r: the taste substance binding induces the conformational change of LBDs so as to shift the relative positions of the C-termini of LBD heterodimers, that induce the positional shift of the CRD regions, and further induce the consequent conformational change of the downstream transmembrane regions for the cytosolic G-protein activation.

The tendency of the conformational change of T1r2a/3LBD observed in solution was similar to those generally observed on crystallographic analyses of other class C GPCR LBDs. Therefore the above presumed scheme for T1r signaling is also basically in line with those for the other class C GPCRs. On the other hand, the manner and extent of the observed conformational change of T1rLBD in the solution are unlikely to resemble to that of GABA_B_R without dimer rearrangement, despite the fact that both T1r and GABA_B_R are heterodimers, but more similar to those of homodimeric mGluR1 with dimer rearrangement.

The VFTD structures were also found in the amino-terminal domain (ATD) of the ionotropic glutamate receptors (iGluRs), one of the extracellular domains serving as a regulatory domain in some receptors. Crystallographic studies of iGluRs-ATD revealed the considerable diversity of the dimer arrangement between the different family members^35^,^36^,^37^. Therefore the VFTD architecture itself likely possesses a characteristic to provide diverse conformational possibilities.

In the case for class C GPCRs, the receptors discussed above have different structural organizations, such that mGluRs, GABA_B_R, and T1r are homodimers with a CRD, a heterodimer lacking a CRD, and a heterodimer with a CRD, respectively. Therefore, the diversity on the conformational equilibriums for the VFTDs (LBDs) might reconcile these structural diversities within the class C GPCRs and be responsible for producing the same consequences at the transmembrane regions: cytosolic G-protein activation. It should be noted that the information for the other class C GPCR LBDs are mostly obtained in the crystalline states without the native glycosyl-chains. Further accumulation of information about the conformations of the member proteins under the conditions closer to the physiological states, including those in the full-length receptors, as well as the high-resolution crystallographic structure information, will be required for understanding the signal transduction mechanisms of the class C GPCR family, and their generalities or diversities.

## Methods

### Expression analysis

The T1r2aLBD and T1r3LBD proteins fused with GFPuv transiently expressed with sf9 cells were analyzed by fluorescence-detection size-exclusion chromatography (FSEC), basically as reported^19^. The samples were loaded either onto a Superose 6 10/300 GL column (GE Healthcare), and the elution profiles were detected using excitation (EX) and emission (EM) wavelengths of 395 nm and 507 nm, respectively. For more details see Supplementary information.

### Measurement of the reactions of T1r2/T1r3 heterodimer to taste substances

The responses of T1r2a/T1r3 to taste substances were analyzed using the Flip-In^TM^ 293 cell line (Life Technologies), which was established for stable expression of T1r2a, T1r3, and Gα16-gust44^38^. The Ca^2+^ flux assays were performed using a FLEX station 3 (Molecular Devices, LLC) for the cells loaded with 100 μl of Hank’s balanced salt solution (Sigma-Aldrich), containing 5 μM of the calcium indicator dye Fluo8 NW (AAT Bioquest).

The conformational change of T1r2a/T1r3LBD upon taste-substance binding was analyzed by Förster resonance energy transfer (FRET) measurement. The T1r2a LBD-Cerulean/T1r3LBD-Venus heterodimer protein was expressed by S2 cells, and purified from the culture medium using ANTI-FLAG M2 Affinity Gel (SIGMA). Fluorescence intensities were recorded at 277 K with a FluoroMax4 spectrofluorometer (Horiba). The sample was excited at 433 nm, and the emission at 526 nm and at 475 nm was recorded, for determination of the FRET index (Intensity at 526 nm/Intensity at 475 nm).

The binding of T1r2a/T1r3LBD to a taste substance was analyzed by isothermal titration calorimetry. T1r2a/3 LBD heterodimer protein was stably expressed by S2 cells, and purified as described above. The purified protein was loaded into the iTC200 cell (GE Healthcare), and the titration was performed at 298 K. For more details see Supplementary information.

### Structure analysis

T1r2a/3 LBD heterodimer protein was stably expressed by S2 cells, and purified from the culture medium using ANTI-FLAG M2 Affinity Gel (SIGMA), followed by size-exclusion chromatography (HiLoad Superdex 200, 16/60 (GE Healthcare)).

The partial specific volume and the molecular weight of the protein were determined by sedimentation velocity analytical ultracentrifugation (SV-AUC) analysis. The samples with same protein concentration but different H_2_O/D_2_O ratios (100% H_2_O, 50% H_2_O/50% D_2_O, and 10% H_2_O/90% D_2_O) were subjected to the SV-AUC analysis performed at 293 K and 42,000 rpm, using a ProteomeLab XL-I analytical ultracentrifuge (Beckman Coulter) with an An-60 Ti rotor. The acquired data were analyzed as reported previously^39^. The specific volume was determined as 0.6787 cm^3^/g.

For electron microscopic observation, the protein sample was applied to a carbon-coated grid and negatively stained with 2% uranyl acetate. The sample grids were examined with a JEM-2100 electron microscope (JEOL) with a LaB_6_ gun operated at an accelerating voltage of 200 kV. Images were recorded on a slow-scan charge-coupled device (SSCCD) camera (MegaScan), at a final magnification of 65,000 and at defocus settings of 8,900 to 27,000 Å. The two dimensional class average of the particles was performed with the EMAN software suite^40^.

SAXS experiments were performed at the SPring-8 beamline BL45XU^41^, or with the Nano-Viewer system (RIGAKU) equipped with a MicroMax-007HF X-ray generator (RIGAKU) with a Cu target (λ = 1.5418 Å) and a PILATUS 200 K detector (DECTRIS). The *R*_g_ values were estimated by the Guinier approximation, using the PRIMUS software^42^. *P*(*r*) functions were calculated by the GNOM software^43^ (Supplementary Table S3). *Ab initio* reconstructions of low-resolution models from the SAXS data were performed by using DAMMIF^44^ (Supplementary Table S4). Multiple reconstructions conducted independently to confirm the reproducibility were scored with the DAMAVER^45^ and DAMCLUST^46^ packages to obtain the most representative models as shown in Fig. 4. For more details see Supplementary information.

## Additional Information

**How to cite this article**: Nango, E. *et al*. Taste substance binding elicits conformational change of taste receptor T1r heterodimer extracellular domains. *Sci. Rep.* **6**, 25745; doi: 10.1038/srep25745 (2016).

## Supplementary Material

Supplementary Information
