# Supplementary material for: Taste substance binding elicits conformational change of taste receptor T1r heterodimer extracellular domains
Source: Sci Rep. 2016 May 10;6:25745. doi: 10.1038/srep25745 (PMC4861910; doi:10.1038/srep25745)
Supplement: Supplementary Information [file srep25745-s1.pdf]

Taste substance binding elicits conformational change of taste receptor T1r heterodimer extracellular domains

Authors: Eriko Nango, Shuji Akiyama, Saori Maki-Yonekura, Yuji Ashikawa, Yuko Kusakabe, Elena Krayukhina, Takahiro Maruno, Susumu Uchiyama, Nipawan Nuemket, Koji Yonekura, Madoka Shimizu, Nanako Atsumi, Norihisa Yasui, Takaaki Hikima, Masaki Yamamoto, Yuji Kobayashi, Atsuko Yamashita

Supplementary Information

Supplementary Methods

Cloning and vector construction

To clone the t1r genes, the lower half portion of the head of a medaka fish, purchased from a local commercial store, was dissected and immediately treated with the TRIZOL reagent (Invitrogen) to isolate the total RNA. The total RNA was reverse transcribed with a PrimeScript 1st strand cDNA Synthesis Kit (Takara), using the oligo dT primer provided by the manufacturer. The T1r genes were amplified from the synthesized first-strand cDNA by KOD Plus Ver.2 DNA polymerase (TOYOBO), using gene-specific primers designed based on the reported fish T1r nucleotide sequences [1].

The ligand binding domains (LBDs) of the T1r2a and T1r3 proteins were assigned based on sequence alignments with that of the metabotropic glutamate receptor 1 used for crystallographic analyses [2,3]. To prepare the expression vectors for fluorescence-detection size-exclusion chromatography (FSEC) [4], pCGFP_SF-mfT1r2aL and pCGFP_SF-mfT1r3L, the genes encoding the LBDs of T1r2a (a.a. 1-474 a.a.) and T1r3 (a.a. 1-491 a.a.) were subcloned into the pCGFP_SF vector [5] between the *Nco*I and *Xho*I sites. To create the expression vectors for the structural and calorimetric analyses, pAc-mfT1r2aL and pAc-mfT1r3L, the genes coding the T1r2aLBD and the T1r3LBD, followed by the Factor Xa cleavage site, the FLAG tag, and the octa-His tag, were subcloned into the pAc5.1/V5-HisA vector (Invitrogen) between the *Eco*RI and *Pme*I sites or the *Kpn*I and *Pme*I sites, respectively. To prepare the expression vectors for Förster resonance energy transfer analyses, pAc-mfT1r2aL-Ce and pAc-mfT1r3aL-Ve, the genes encoding T1r2aLBD followed by Cerulean (Ce, a blue fluorescent protein variant for the donor) and the FLAG-tag, or the T1r3LBD followed by Venus (Ve, a yellow fluorescent protein variant as the acceptor) and the FLAG tag [6], were subcloned into the pAc5.1/V5-HisA vector in the same manner as

pAc-mfT1r2aL and pAc-mfT1r3L.

Expression analysis by fluorescence-detection size-exclusion chromatography (FSEC)

The expression of T1r2aLBD and T1r3LBD was analyzed by conventional FSEC (single-labeled FSEC) as described previously [5]. Briefly, Sf9 cells were transfected with pCGFP_SF-mfT1r2aL and/or pCGFP_SF-mfT1r3L, using Insect Genejuice (Novagen) for transient expression. After 48 hours, the cells and the culture medium were collected separately. The supernatant of the cell lysate, prepared by sonication, and the concentrated medium were each loaded onto a Superose 6 10/300 GL column (GE Healthcare). The elution profiles were detected with a fluorometer, RF-10AXL (Shimadzu), using an excitation wavelength of 395 nm and an emission wavelength of 507 nm.

Measurement of the response of T1r2 and T1r3 to taste substances

The full-length mfT1r2a and T1r3 genes were subcloned into the pEF-DEST51 (Life Technologies) and pEAK10 (Edge BioSystems) vectors, respectively, for expression in cultured cells. Gα16-gust44 expressing cells were used for coupling T1r2 and T1r3. The Gα16-gust44 construct, described previously [7], was obtained by overlapping PCR and subcloned into the pcDNA5/FRT vector (Life Technologies). The responses of T1r2a/T1r3 to taste substances were analyzed using the Flip-In™ 293 cell line (Life Technologies), cultured in modified Dulbecco's modified Eagle's medium with GlutaMax (Life Technologies) and 10% dialyzed-FCS (Life technologies), to avoid T1r2/T1r3 internalization by stimulation from amino acids in the culture medium. Gα16-gust44 was transfected into Flip-In™ 293 cells to generate a cell line that stably expressed the Gα16-gust44 protein (G16-gust44 cells) prior to transfection with T1r2a and T1r3. A stable cell line expressing T1r2a and T1r3 was then generated by transfecting the Gα16-gust44 cells with the corresponding plasmid vectors. Cells were selected for antibiotic resistance to hygromycin (InvivoGen, 100 μg/mL) for Gα16-gust44, blasticidin (InvivoGen, 10 μg/mL) for T1r2a, and puromycin (InvivoGen, 1 μg/mL) for T1r3. Resistant colonies were expanded, and used for 96-well plate calcium (Ca^{2+}) flux assays. The Ca^{2+} flux assays were performed using a FLEX station 3 (Molecular Devices, LLC). Cells from the stable cell line (2.5×10^4) were transferred into a glass-based 96-well plate (Griner Bio One, Frickenhausen, Germany). After 16–30 h, the wells were loaded with 100 μl of Hank's balanced salt solution (Sigma-Aldrich), containing 5 μM of the calcium indicator dye Fluo8 NW (AAT Bioquest), for 60 min at 37°C. The stimulation was

performed by adding 25 μl of 5× concentrated solutions of taste substances, using a pipette. The intensity of the response was represented as the ratio relative to the maximum response value of T1r2a/T1r3 to alanine, and was plotted versus the ligand concentration. The concentration-response curves were fitted to the Hill equation, using the SigmaPlot software (Systat Software).

Protein preparation

The expression vectors pAc-mfT1r2aL, pAc-mfT1r3L, and pCoBlast (Invitrogen) were transfected into S2 cells by the calcium phosphate transfection method, according to the manufacturer's protocol (Invitrogen). S2 cells stably and constitutively expressing both mfT1r2aL and mfT1r3L were selected and cloned in soft agar, consisting of Schneider's *Drosophila* medium (Invitrogen), 10 % fetal bovine serum (FBS), 25 μg/mL blasticidin S, and 0.3 % low melting point agarose, at 300 K. The detailed protocol for the selection process will be published elsewhere. The selected stable clone was adapted to suspension culture in ExpressFive SFM (Invitrogen) containing 10 μg/mL blasticidin S, at 300 K. Five days prior to protein purification, the cells were centrifuged and resuspended in fresh ExpressFive SFM, and cultured further at 300 K.

All of the following purification procedures were performed at 277 K. The culture medium was collected after the removal of the cells by centrifugation, and was supplemented with PMSF, L-alanine, and $CaCl_2$ to final concentrations of 0.5 mM, 0.1 M, and 40 mM, respectively. The pH of the medium was then adjusted to 8 by adding a solution of 1 M Tris-HCl. After an incubation for 60 min, the medium was centrifuged and filtered with a VacuCap 90 (Pall). The mfT1r2a/3 LBD protein in the medium was bound to ANTI-FLAG M2 Affinity Gel (SIGMA) by continuous circulation through the column packed with the resin overnight. The FLAG resin was washed with 120 column volumes of buffer A (20 mM Tris, 0.1 M L-alanine, 2 mM $CaCl_2$, 0.3 M NaCl, pH 8.0), and the protein was eluted with 5 column volumes of buffer A containing 100 μg/mL FLAG peptide. The protein concentration was estimated by absorbance at 280 nm with Nanodrop (Thermo Scientific), using the extinction coefficient calculated from the amino acid sequences (Abs_{280nm}(1 mg/mL) = 1.321), unless otherwise stated.

Sedimentation velocity analytical ultracentrifugation (SV-AUC) analysis

The purified mfT1R2a/3 LBD protein was dialyzed against buffer B (20 mM Tris, 5

mM Gln, 2 mM $CaCl_2$, 0.3 M NaCl pH 8.0) overnight, followed by further purification on a HiLoad 16/600 Superdex 200 pg column (GE HealthCare) in buffer B. To determine the partial specific volume and the molecular weight of the protein from the SV-AUC experiments, samples with same protein concentration but different H_2O/D_2O ratios were prepared. The sample concentration was adjusted so that the absorbance at 280 nm was approximately 0.85 OD, in buffer B with three different H_2O/D_2O ratios: 100 % H_2O, 50 % H_2O/50 % D_2O, and 10 % H_2O/90 % D_2O. The pD for buffer B prepared using D_2O was adjusted by assuming pD = pH meter reading + 0.40 [8]. Buffer densities were measured using a DMA 4500M densitometer (Anton Paar), and viscosity measurements were performed with a Lovis 2000 M/ME viscometer (Anton Paar). The SV-AUC experiment was performed at 293 K and 42,000 rpm, using a ProteomeLab XL-I analytical ultracentrifuge (Beckman Coulter) with an An-60 Ti rotor. The acquired data were analyzed as reported previously [9], using the SEDPHAT “hybrid global continuous distribution and global discrete species” model [10].

Förster Resonance Energy Transfer (FRET)

The mfT1r2aL-Ce and mfT1r3L-Ve vectors were co-transfected into S2 cells by the calcium phosphate transfection method, in Schneider’s *Drosophila* medium containing 10 % FBS at 300 K, according to the manufacturer’s protocol (Invitrogen). One day later, the transfected cells were washed with and resuspended in fresh medium, and further incubated for 5 days at 300 K. The transiently-expressed heterodimer protein was purified as described in the “Protein preparation” section. The purified protein was dialyzed against buffer C (20 mM Tris, 10 mM alanine, 2 mM $CaCl_2$, 0.3 M NaCl, pH 8.0) overnight. After concentration and ultracentrifugation, the protein was diluted with buffer C containing various concentrations of alanine or glutamine. Each sample was incubated at 277 K overnight and subjected to fluorescence measurement.

Fluorescence spectra were recorded at 277 K with a FluoroMax4 spectrofluorometer (Horiba). The sample was excited at 433 nm, and FRET was detected by the emission at 526 nm. The emission at 475 nm was also recorded for the FRET index calculation. The FRET index (Intensity at 526 nm/Intensity at 475 nm) was plotted against the ligand concentration, and the titration curves were fitted to the Hill equation by using KaleidaGraph (Synergy Software).

Isothermal titration calorimetry

The purified mfT1r2a/3 LBD heterodimer was extensively dialyzed against buffer D (20 mM Tris, 0.3 M NaCl, 2 mM $CaCl_2$, pH 8.0) at 277 K overnight. The dialyzed protein solution (~49 μM) was loaded into the iTC200 cell (GE Healthcare). The titration was performed by injecting 2 μL of the syringe solution (0.72 mM L-glutamine or 2 mM L-alanine) at intervals of 120 s at 298 K. The thermograms and the binding isotherms were analyzed with NITPIC [11] and SEDPHAT [10], respectively. We performed the fitting assuming that one ligand binds to one heterodimer, and included the correction factors for the protein concentration for fitting.

Electron microscopy

The protein sample was prepared as described in the previous section. A few microliters of 0.05 to 0.1 mg/mL purified protein solution were applied to a carbon-coated grid and negatively stained with 2% uranyl acetate. The sample grids were examined with a JEM-2100 electron microscope (JEOL) with a LaB_6 gun operated at an accelerating voltage of 200 kV. Images were recorded on a slow-scan charge-coupled device (SSCCD) camera (MegaScan), at a final magnification of 65,000 and at defocus settings of 8,900 to 27,000 Å. The magnification was calibrated from catalase crystals. We manually picked particles from 150 EM images of each sample, by using the boxer program[12]. The set of particles was classified and averaged with the refine2D program based on iterative MSA-based particle classification and averaging from the EMAN software suite [12]. The 2d class averages was performed with 21,663 molecular images in the L-glutamine-bound form and 10,005 molecular images in the ligand free form by ten iterations.

Small-angle X-ray scattering (SAXS)

The sample of the L-glutamine-bound form was prepared as described in the sections "Protein preparation" and "Sedimentation velocity analytical ultracentrifugation (SV-AUC) analysis". For the sample of the ligand-free form, the purified protein was dialyzed twice against buffer C and once against buffer D, and then was subjected to chromatography on a Superdex 200 column in buffer D. The purified protein sample was concentrated to ~9 mg/mL, and five different concentrations of the protein sample (3.4 ~ 9.0 mg/mL) were prepared by dilution. BSA (66.4 kDa [13], Sigma) was used as the standard protein [41].

SAXS experiments were performed at the SPring-8 beamline BL45XU, or with the Nano-Viewer system (RIGAKU) equipped with a MicroMax-007HF X-ray generator (RIGAKU) with a Cu target (λ = 1.5418 Å) and a PILATUS 200K detector (DECTRIS). The

data collected at a camera distance of 699 mm at 283 K were normalized by the exposure time (30 min) and the protein concentration. The R_g values were estimated by the Guinier approximation, using the PRIMUS software [14]. $P(r)$ functions were calculated by the GNOM software [15].

Structural analyses

The dimerization manners were evaluated with the torsion angles between the axes running through the two protomers, which connect the codebook vectors of LB1 and LB2 in each protomer (Fig. 1a), based on the vector quantization performed by QPDB in the Situs package [16,17]. The estimated R_g values and the envelope diameters for PDB files were calculated using CRYSOL [18]. The theoretical $P(r)$ functions of high-resolution structures were estimated using CRYSOL and GNOM [15]. In all cases, the common regions included in the coordinate files, and ligand molecules if bound, but without water and other heteroatom entries, were used for the calculation, in order for accurate comparison.

Ab initio reconstructions of low-resolution models from the SAXS data were performed by using a DAMMIF package [19]. The models were restored under an assumption of two-fold symmetry, because the two-dimensional averages of T1r2a/3LBD particles suggested a quasi-twofold symmetric shape (Figure 2). A slight weight on oblate shapes resulted in more reproducible reconstructions in the case of the ligand-free state. Multiple reconstructions were conducted independently and were scored with the DAMAVER [20] and DAMCLUST [21] packages to obtain the most representative models as shown in Figure 4. The statistics for the model reconstructions were summarized in Supplementary Table S4. The representative models were presented as smooth molecular envelopes using the SITUS package [16,17], onto which known high-resolution models were superimposed using the SUPCOMB package [22]. The structural figures were prepared with PyMol (Schrödinger).

References for Supplementary Information

1 Ishimaru, Y. *et al.* Two families of candidate taste receptors in fishes. *Mech Dev* **122**, 1310-1321 (2005).

2 Kunishima, N. *et al.* Structural basis of glutamate recognition by a dimeric metabotropic glutamate receptor. *Nature* **407**, 971-977 (2000).

3 Tsuchiya, D., Kunishima, N., Kamiya, N., Jingami, H. & Morikawa, K. Structural views of the ligand-binding cores of a metabotropic glutamate receptor complexed with an antagonist and both glutamate and Gd3+. *Proc Natl Acad Sci U S A* **99**,

2660-2665 (2002).
4 Kawate, T. & Gouaux, E. Fluorescence-detection size-exclusion chromatography for precrystallization screening of integral membrane proteins. *Structure* **14**, 673-681 (2006).
5 Ashikawa, Y. *et al.* GFP-based evaluation system of recombinant expression through the secretory pathway in insect cells and its application to the extracellular domains of class C GPCRs. *Protein Sci* **20**, 1720-1734 (2011).
6 Koushik, S. V., Chen, H., Thaler, C., Puhl, H. L., 3rd & Vogel, S. S. Cerulean, Venus, and VenusY67C FRET reference standards. *Biophys J* **91**, L99-L101 (2006).
7 Ueda, T., Ugawa, S., Yamamura, H., Imaizumi, Y. & Shimada, S. Functional interaction between T2R taste receptors and G-protein alpha subunits expressed in taste receptor cells. *J Neurosci* **23**, 7376-7380 (2003).
8 Glasoe, P. K. & Long, F. A. Use of glass electrodes to measure acidities in deuterium oxide. *J. Phys. Chem.* **64**, 188-190 (1960).
9 Brown, P. H., Balbo, A., Zhao, H., Ebel, C. & Schuck, P. Density contrast sedimentation velocity for the determination of protein partial-specific volumes. *PLoS One* **6**, e26221 (2011).
10 Houtman, J. C. *et al.* Studying multisite binary and ternary protein interactions by global analysis of isothermal titration calorimetry data in SEDPHAT: application to adaptor protein complexes in cell signaling. *Protein Sci* **16**, 30-42 (2007).
11 Keller, S. *et al.* High-precision isothermal titration calorimetry with automated peak-shape analysis. *Anal Chem* **84**, 5066-5073 (2012).
12 Ludtke, S. J., Baldwin, P. R. & Chiu, W. EMAN: semiautomated software for high-resolution single-particle reconstructions. *J Struct Biol* **128**, 82-97 (1999).
13 Hirayama, K., Akashi, S., Furuya, M. & Fukuhara, K. Rapid confirmation and revision of the primary structure of bovine serum albumin by ESIMS and Frit-FAB LC/MS. *Biochem Biophys Res Commun* **173**, 639-646 (1990).
14 Konarev, P. V., Volkov, V. V., Sokolova, A. V., Koch, M. H. J. & Svergun, D. I. PRIMUS: a Windows PC-based system for small-angle scattering data analysis. *Journal of Applied Crystallography* **36**, 1277-1282 (2003).
15 Svergun, D. I. Determination of the Regularization Parameter in Indirect-Transform Methods Using Perceptual Criteria. *Journal of Applied Crystallography* **25**, 495-503 (1992).
16 Wriggers, W., Milligan, R. A., Schulten, K. & McCammon, J. A. Self-organizing

neural networks bridge the biomolecular resolution gap. *J Mol Biol* **284**, 1247-1254 (1998).

17 Wriggers, W. Using Situs for the integration of multi-resolution structures. *Biophys Rev* **2**, 21-27 (2010).

18 Svergun, D., Barberato, C. & Koch, M. H. J. CRYSOL - A program to evaluate x-ray solution scattering of biological macromolecules from atomic coordinates. *Journal of Applied Crystallography* **28**, 768-773 (1995).

19 Franke, D. & Svergun, D. I. DAMMIF, a program for rapid ab-initio shape determination in small-angle scattering. *Journal of Applied Crystallography* **42**, 342-346 (2009).

20 Volkov, V. V. & Svergun, D. I. Uniqueness of ab initio shape determination in small-angle scattering. *Journal of Applied Crystallography* **36**, 860-864 (2003).

21 Petoukhov, M. V. *et al.* New developments in the ATSAS program package for small-angle scattering data analysis. *Journal of Applied Crystallography* **45**, 342-350 (2012).

22 Kozin, M. B. & Svergun, D. I. Automated matching of high- and low-resolution structural models. *Journal of Applied Crystallography* **34**, 33-41 (2001).

23 Muto, T., Tsuchiya, D., Morikawa, K. & Jingami, H. Structures of the extracellular regions of the group II/III metabotropic glutamate receptors. *Proc Natl Acad Sci U S A* **104**, 3759-3764 (2007).

24 Geng, Y., Bush, M., Mosyak, L., Wang, F. & Fan, Q. R. Structural mechanism of ligand activation in human GABA(B) receptor. *Nature* **504**, 254-259 (2013).

Supplementary Table S1. Representative crystal structures of the extracellular domains of class C GPCRs.

protein	ligand	ligand type	dimer arrange.[*]	protomer structure[†]	Torsion angle[‡] (˚)	C-term distance[§] (Å)	est. R_g [ǀ] (Å)	Envelope d. [‖] (Å)	PDB ID	Ref.
mGluR1	Glu	agonist	A	C-O	-6.99	66.0	30.8	98.29	1EWK	1
mGluR1	–	–	R	O-O	-58.13	84.4	33.9	111.4	1EWT	1
mGluR1	–	–	A	C-O	-4.58	68.4	31.0	99.81	1EWV	1
mGluR1	Glu, Gd^{3+}	agonist	A	C-C	-25.27	64.2[☆]	30.4[☆]	97.57	1ISR	2
mGluR1	MCG	antagonist	R	O-O	-57.93	86.7	33.8	110.5	1ISS	2
mGluR1	LY341495	antagonist	A	O-O	-0.95	71.6	31.4	100.3	3KS9	[**]
mGluR3	Glu	agonist	R	C-C	–	–	–		2E4U	3
mGluR3	LY341495	antagonist	R	O-O	–	–	–		3SM9	[**]
mGluR5	Glu	agonist	A	C-C	–	–	–		3LMK	[**]
mGluR7	LY341495	antagonist	R	O-O	–	–	–		3MQ4	[**]
GABA$_B$R[¶]	–	–	A[#]	O-O	-22.78	45.5	31.3	95.71	4MQE	4
GABA$_B$R[¶]	GABA	agonist	A[#]	C-O	-22.64	32.0	29.4	92.12	4MS3	4
GABA$_B$R[¶]	CGP54626	antagonist	A[#]	O-O	-25.61	44.8	31.0	95.34	4MR7	4

[*]Dimer arrangement observed in the crystal structure. A: "active" orientation, R: "resting" orientation. [†]Protomer conformation observed in the crystal structure. O: open state, C: closed state. OO or CC indicates that both protomers adopt the open or closed state, respectively, while CO indicates that one protomer adopts the closed state, while the otheradopts the open state. [‡]The torsion angle between the axes running through the two protomers, which connect the codebook vectors of LB1 and LB2 in each protomer (Fig. 1a in the main text), based on the vector quantization performed by QPDB in the Situs package [16,17]. [§]Distance between the C*a* atoms of the C-terminal residue in each protomer. For mGluR1, those between D509 in each protomer are shown, while for GABA$_B$R, those between D459 in GBR1 and D466 in GBR2 are shown. [ǀ]Estimated by CRYSOL [18]. For mGluR1, the coordinates in the range of R36-D509 and ligand molecules if bound, but without water and other heteroatom entries, were used for the calculation. For GABA$_B$R, the coordinates in the range of R50-D459 in GBR1 and those in the range of S53-D466 in GBR2 were used for the calculation. [‖]Envelope diameter, which corresponds to the maximum dimension in a protein molecule, estimated by CRYSOL [18]. Other settings are same as estimated R_g calculation. [¶]GABA$_B$R receptor GBR1/GBR2 heterodimer. [#]The dimer arrangement was defined by the similarity to either the A- or R-orientation observed in mGluR structures, while in the original report, the terms "active" and "resting" were defined in terms of physiological states. [☆]The crystal structure was solved with one protomer in an asymmetric unit, and the characterizations were made using the two protomers related by crystallographic 2-fold symmetry. [**]Unpublished. 1[2]. 2 [3]. 3 [23]. 4 [24].

Supplementary Table S2. EC_{50} values and the binding constants for the T1r2a and T1r3 heterodimer to taste substances *

EC$_{50}$ values by the responses on the mfT1r2a/3 full-length receptor

Taste substance	EC_{50} (μM)
L-glutamine	100 ± 26.0
L-alanine	2700 ± 1200

EC$_{50}$ values for the FRET signal changes of mfT1r2a/3LBD

	EC_{50} (μM)
L-glutamine	12.7 ± 2.7
L-alanine	168 ± 19.0

Dissociation constant to the mfT1r2a/3LBD, analyzed by isothermal titration calorimetry†

Taste substance	K_d (μM)	ΔH (kcal/mol)	Correction factor‡
L-glutamine	8.9	-13.1	0.232
L-alanine	43.2	-4.0	0.169

*The values shown are means ± SEM of n independent determinations, where n = 4−34 for the receptor response assay, and n = 3 for the FRET measurement. †Fitting result assuming 1 ligand: 1 heterodimer binding. ‡Correction factor for the protein concentration.

Supplementary Table S3. SAXS structural parameters.

	R_g * (Å)	R_g † (Å)	$I(0)$ * (a.u.)	$I(0)$ † (a.u.)	D_{max} ‡ (Å)	V_p § ($Å^3$)	*MM* from $I(0)$ ‖ (kDa)	*MM* from V_p ¶ (kDa)	*MM* g (kDa)
Ligand-free	39.8 ± 0.6	41.4 ± 0.2	101.7 ± 0.9	102.6 ± 0.5	143	2.47×10^5	123 ± 1	150	127 #
L-Gln-bound	37.0 ± 0.5	35.7 ± 0.1	100.4 ± 0.8	97.9 ± 0.4	112	2.38×10^5	121 ± 1	144	(109 ☆)
BSA	31.6 ± 0.4	n.d.	55.1 ± 0.4	n.d.	n.d.	n.d.	n.d.	n.d.	66.4[13]

* Guinier analysis using the Q range from 0.01003 to $Q_{max} < 1.3 / R_g$. † Estimates in real space upon $P(r)$ determination. ‡ Maximum dimension estimated by using GNOM package. § Porod Volume. ‖ Molecular mass calculated by using the $I(0)$ value for BSA as the standard. ¶ Molecular mass calculated according to an empirical relationship ($MM = V_P / 1.65$, [21]). # The molecular mass of the T1r2a/3LBD heterodimer estimated from SDS-PAGE and mass spectrometry (Supplementary Fig. S1). ☆ The molecular mass of the T1r2a/3LBD heterodimer estimated from the amino acid sequence, without assuming posttranslational modification.

Supplementary Table S4. SAXS shape-reconstruction statistics for T1r2a/3LBD heterodimers

	L-Gln-bound State	Ligand-free State
Shape Reconstruction	DAMMIF	DAMMIF
Q range (Å^{-1})	0.01003 - 0.2006	0.01003 - 0.2006
Symmetry	*P*2	*P*2
Weighting on Particle Asymmetry	none	oblate
Number of spherical harmonics	20	20
Number of Shannon channels	8	8
SQRT(χ^2) (mean ± S.D.)	1.718 – 1.745 (1.733 ± 0.006)	1.694 – 1.761 (1.726 ± 0.019)
Total Number of Reconstructions	50	50
Number of Models Averaged (best cluster from DAMCLUST)	26	24
DAMAVER NSD (mean ± S.D.)	0.590 – 0.737 (0.641 ± 0.043)	0.590 – 1.269 (0.736 ± 0.130)

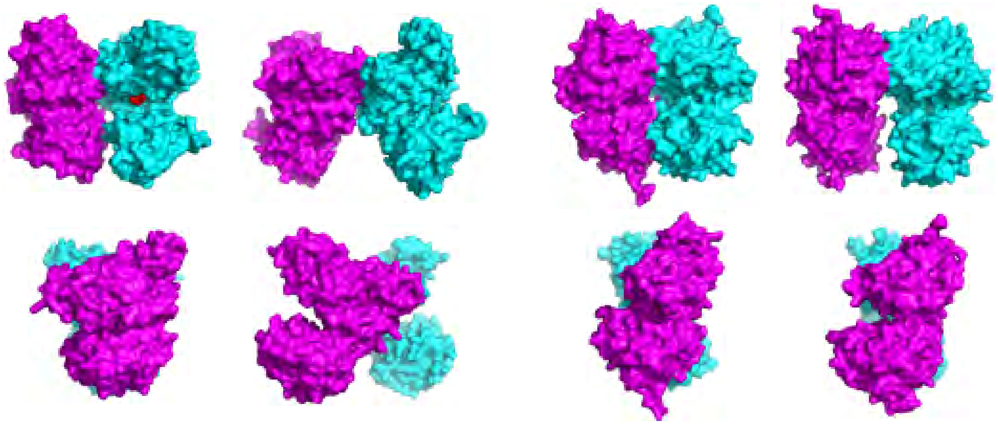

closed-open/A-state
mGluR1, Glu-bound
(1EWK)

open-open/R-state
mGluR1, ligand-free
(1EWT)

closed-open/A-state
GABA$_B$R, GABA-bound
(4MS3)

open-open/A-state
GABA$_B$R, ligand-free
(4MQE)

Supplementary Figure S1. Representative crystal structures of class C GPCR LBD. The surface of each protomer was colored in either magenta or cyan. For GABAB receptor structures, the ligand-binding protomer, GABABR1 was colored in magenta, while the non-ligand-binding protomer GABABR2 was colored in cyan. The bound ligands were shown as CPK models in red. “Open” or “closed” indicates the protomer conformation in terms of the domain closure, and “A” or “R” indicates the dimerization manner, as described in the text.

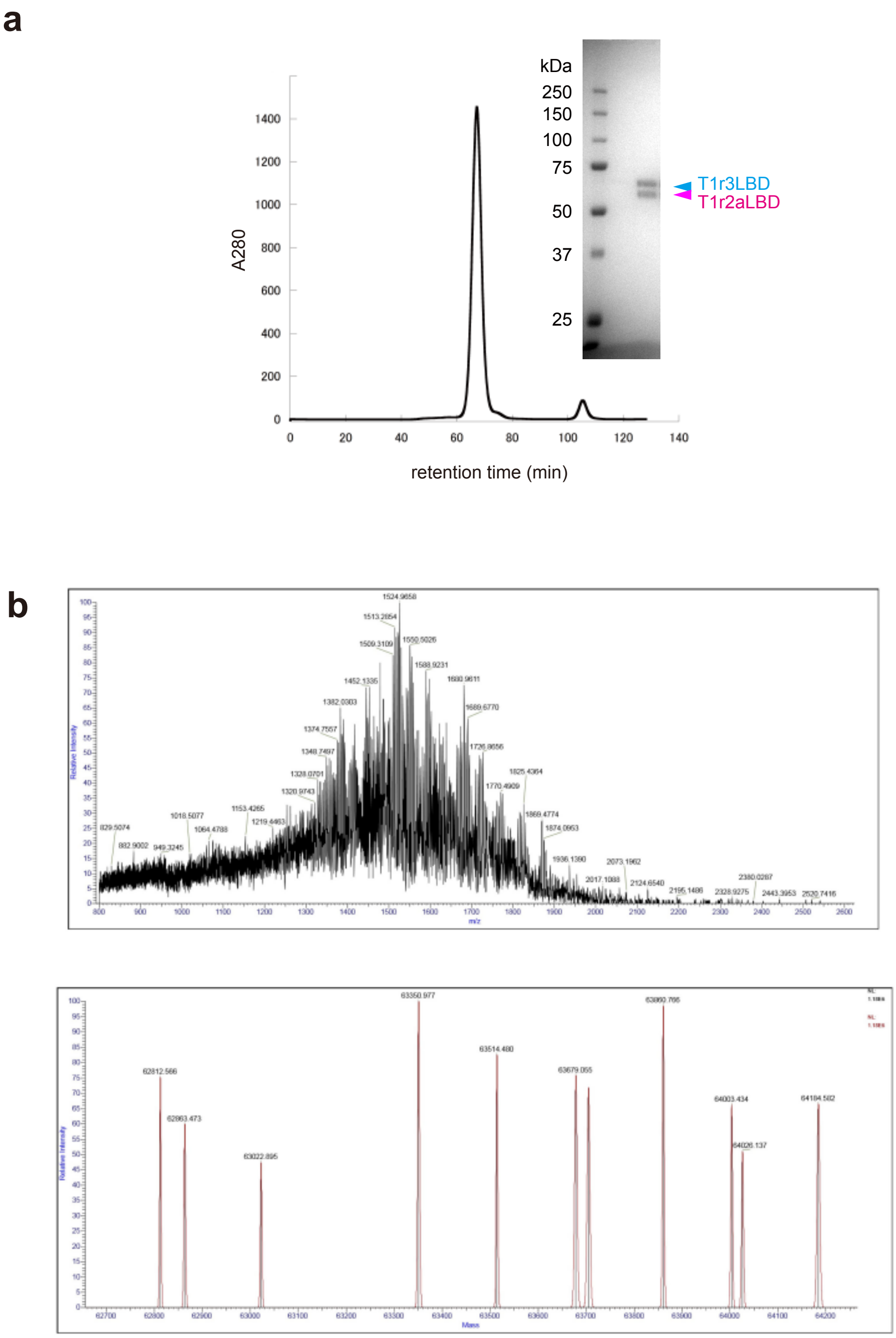

Supplementary Figure S2. Characterization of the Recombinant T1R2a/3LBD Heterodimer from medaka fish. (a) SEC profile of the T1R2a/T1R3 LBD heterodimer, in a large-scale preparation. The final SDS-PAGE result is shown in the inset. Based on the migrations of the T1r2aLBD and T1r3LBD protein bands, the molecular weights were estimated about 60 kDa and 67 kDa, respectively. (b) Mass spectroscopic analysis of theT1R2a/3LBD heterodimer. The top two observable peaks were 63,350 and 63,674 Da. The observed high heterogeneity of the mass peaks as well as the larger masses than those estimated from the amino acid sequences indicate that the protein sample is glycosylated.

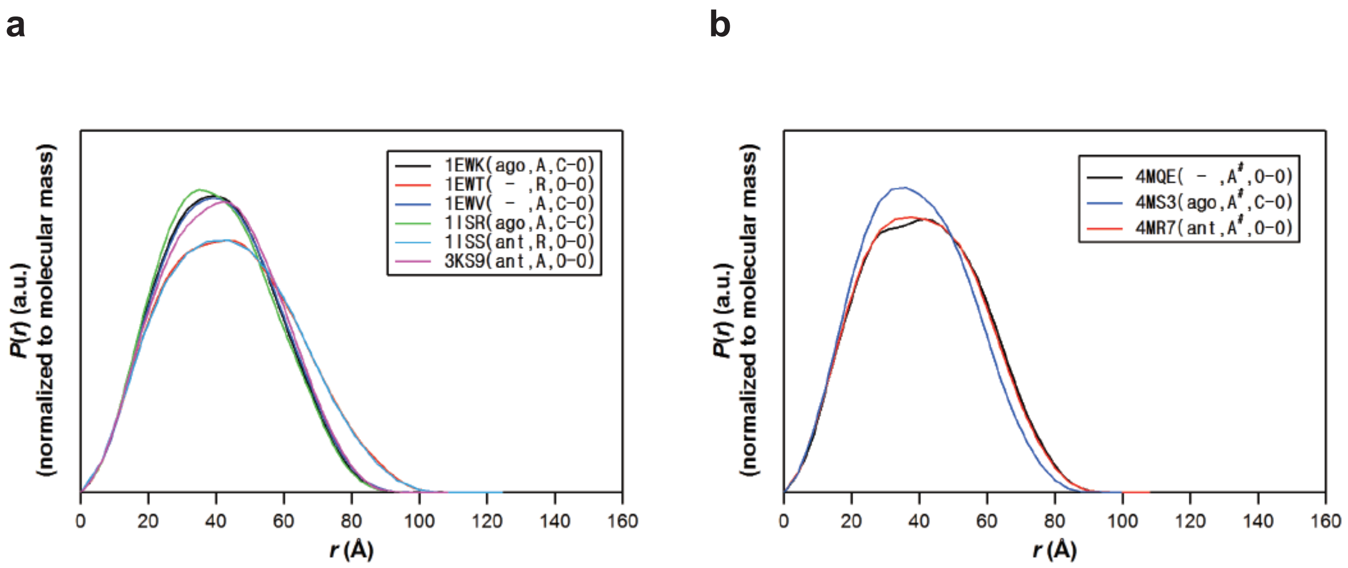

Supplementary Figure S3. Pair distribution functions, $P(r)$, calculated from the crystal structures of the class C GPCR LBDs. (a) mGluR1. (b) GABABR. The coordinate files used for the calculation were the same as described in the footnote in Supplementary Table S1.
